# Seasonal Variations in Physical Activity Domains among Rural and Urban Bangladeshis Using a Culturally Relevant Past Year Physical Activity Questionnaire (PYPAQ)

**DOI:** 10.1155/2019/2375474

**Published:** 2019-10-13

**Authors:** Shirin Jahan Mumu, Paul P. Fahey, Liaquat Ali, A. K. M. Fazlur Rahman, Dafna Merom

**Affiliations:** ^1^School of Science and Health, Western Sydney University, Sydney 2751, Australia; ^2^Department of Epidemiology, Bangladesh University of Health Sciences (BUHS), Dhaka 1216, Bangladesh; ^3^Pothikrit Centre for Health Studies, Dhaka 1000, Bangladesh

## Abstract

While the effect of weather and seasons on physical activity (PA) is well documented for leisure-time physical activities in western countries, scant information is available for developing countries where lifestyle PA is the major source of energy expenditure (EE). In Bangladesh, the traditional calendar divides the year to six seasons that last two months each: summer, rainy, autumn, late autumn, winter, and spring. We developed the Past Year Physical Activity Questionnaire to record culturally relevant physical activities and to help assess the seasonal variation in total and domain-specific PA in Bangladesh. We have applied this tool to 162 men and women aged 18–60 years residing in Dhaka city and in the northern rural district of Thakurgaon. Repeated measures analysis of variance (RMANOVA) was used to test for evidence of variation in PA between place and seasons. The age- and gender-adjusted model revealed significantly lower levels of EE in urban residents compared to rural residents across all seasons and domains. We also found evidence of seasonal variations in moderate-to-vigorous physical activity (MVPA) MET-min/weekamong rural participants only; for total PA (ranging from 3192 in autumn to 4124 in winter; *p* = 0.0001) and for two domains: the occupation domain (ranging from 935 in autumn to 1645 in winter; *p* = 0.0001) and the leisure time domain(ranging from 229 in late autumn to 272 in rainy season; *p* = 0.005). Seasonality in gardening was also noted (ranging from 2.46 in late autumn to 29.28 in rainy season; *p* = 0.0001). There were no seasonal differences of total and domain-specific MVPA in urban except household-related PA. Among rural participants, PA was higher in the summer, rainy, and winter seasons and lower in autumn and late autumn. The most common leisure-time physical activities were walking, bicycling, and swimming with higher participation in the rural area. Leisure-time physical activity needs to be promoted to urban residents all year long but more focused on autumn, late autumn, and spring in rural areas.

## 1. Introduction

In recent years, there has been increasing recognition of the role of physical activity (PA) in primary and secondary prevention of noncommunicable diseases (NCDs). This has occurred in both high- and low-income countries [1–3]. It has been estimated that at least 9% of premature mortality globally could be avoided if everyone adhered to the PA guidelines of the World Health Organization (WHO) [4]. Hence, monitoring and promoting PA is a public health priority.

The role of the man-made environment in shaping PA patterns has been extensively studied in the past two decades, particularly in relation to urban design. However, the impact of season, weather, geography, and culture on PA is less well known [5]. A review of the international literature on PA seasonality and regional variations by Tucker and Gilliland highlighted that weather is a barrier for PA participation and that both adults and children are generally more active in the summer than during the winter [5]. This review relied on data from eight different studies, all, with the exception of one study of Guatemalan children, conducted in developed countries (USA, Canada, UK, France, Netherlands, and Australia). Hence, the accumulated knowledge of seasonal variations in PA mostly pertains to leisure-time physical activity (LTPA); a domain that contributes significantly to total PA in the western world [6, 7]. Little is known about seasonal variations in PA in developing countries, such as Bangladesh, where lifestyle PA is the major contributor to overall PA. The studies which have been conducted in Bangladesh have shown that less than 15% of the population participates in any moderate-to-vigorous LTPA and that occupation and travel were the major contributors to total PA in both urban and rural areas [2, 3]. The impact of season is not yet documented.

Traditionally, Bangladeshis subdivide the year into six seasons that follow the agricultural cycle. Each season lasts two months. The year begins with summer (from mid-April to mid-June), followed by the rainy season (mid-June to mid-August), autumn (mid-August to mid-October), late autumn (mid-October to mid-December), winter (mid-December to mid-February), and spring (mid-February to mid-April) [8]. However, in terms of weather conditions, three main seasons are distinguishable: a hot, humid summer, or premonsoon from March to June; a cool, rainy monsoon season from June to October; and a cool, dry winter from October to March. Further, given the more traditional rural lifestyle and the more westernized urban lifestyle, the impact of seasons on PA may differ between rural and urban residents. This makes Bangladesh an interesting place to explore seasonality. In order to better measure the PA pattern in Bangladesh and to inform future intervention strategies, we have developed the Past Year Physical Activity Questionnaire (PYPAQ), which assesses culturally relevant moderate-to-vigorous physical activities (MVPA) categorized into key domains and incorporating each season. The aims of this study were to: (1) test for seasonal variation in total energy expenditure and in each PA domain; (2) compare seasonal variation in average PA between a group of rural residents and a group of urban residents.

## 2. Materials and Methods

### 2.1. Participants and Data Collection

Ethics approval was obtained from the Western Sydney University Human Research Ethics Committee (HREC # H11145) and the Bangladesh University of Health Science Ethical Review Committee.

Participants in this study were recruited for a validation study that is fully described elsewhere [9]. Briefly, a group of rural residents were selected from Satia village of Pirganj Subdistrict of Thakurgaon District and a group of urban residents from the Bangladesh University of Health Sciences (BUHS), which is situated in the north of Dhaka city. Urban participants were recruited conveniently from twelve distinct occupations ranging from professor to cleaner. For the rural sample, each household (HH) of Satia village was approached starting at the nearest house on the left side of the main road and then moving on to the next-nearest HH until the sample size was reached. From each HH, only one eligible person was selected. The inclusion criteria for both places were that participants had to be of either gender, aged between 18 and 60 years and generally healthy. We excluded those who were physically disabled or suffering from any chronic medical condition, which limited their physical activity; including mental retardation. We have asked the potential participants or their relatives about any medical condition that they had. If they had any medical records, we checked those for confirmation. We excluded pregnant women as this may also affect pattern of PA.

### 2.2. Past Year Physical Activity Questionnaire (PYPAQ) Development

We used the Minnesota Leisure Time Physical Activity (MLTPA) Questionnaire [10] as a template for this questionnaire. In particular, we followed its recall time frame that allows the recording of seasonal changes in PA. In the MLTPA Questionnaire, the respondent is asked to report the frequency and amount of time spent on each PA over the previous year on a month-by-month basis. The list of types of PA people who could participate in is extensive, and each is assigned a PA intensity code or Metabolic Equivalent Tasks (METs). The PA intensity codes were derived from the Physical Activity Compendium [10, 11].

We have changed the list of activities to match the Bangladesh culture. The activities are grouped into five domains: occupation; transport; household; gardening; and leisure-time activities. Within the occupation category, there are six subcategories: farming; driving; day laboring; tailoring; office work (i.e., doctor, teacher, and executive); and “other.” The questionnaire assesses PA over the previous year covering all six seasons [8]. Trained interviewers asked respondents for detail about their physical activities during the past 12 months and recorded comprehensive information on each activity undertaken. For example, participants were asked to specify the months in which the activity was performed, the usual frequency per month, and the number of hours spent per occasion. The questionnaire captures all PA including paid employment and gathers detailed information on time spent sitting, standing, and walking while at work.

### 2.3. PYPAQ Outcome Measures

Using the Compendium of PA, we first assigned the appropriate MET value to each activity reported [12]. A MET is the ratio between the energy expenditure while performing the activity relative to energy expenditure when in a resting position (1 MET). We considered METs from 3.0 to 6.0 as moderate activity and greater than 6.0 as vigorous activity. For example, the intensity code of brisk walking is 3.8 METs, which suggests that this activity is a moderate-intensity activity and has 3.8 times higher energy expenditure than resting on average.

The MET-hours per year for each activity for each respondent was calculated by multiplying the MET for that activity with the number of months when the activity was performed, the average number of occasions per month that the activity was performed and the average duration per occasion. Each participant's domain-specific PAs were obtained by summing the MET-hours per year of all activities listed in that domain (e.g., occupation, transport, and household). Finally, results were converted to MVPA MET-hours per week (by dividing by 52) and MVPA MET-minutes per week for analysis and presentation.

We validated PYAPQ against the Global Physical Activity Questionnaire (GPAQ) and concurrent validity for total MVPA was 0.42. Spearman's correlation between the GPAQ measure of usual week MVPA and the PYPAQ average weekly MVPA was 0.61 and 0.39 for urban and rural residents, respectively.

### 2.4. Statistical Analysis

The sample size was determined by the needs of a previous analysis. However, we had designed a new study of this type where the current sample size would allow at least 80% power to detect a statistically significant (*p*<0.05) decrease in the rural group of at least 0.30 standard deviation of the mean MVPA in two seasons (relative to the other four), assuming a conservative 0.2 correlation in mean MVPA between seasons. An equivalent 0.38 or more standard deviation change in the urban group would have at least 80% power of being detected as statistically significant and a 0.45 or more standard deviation change in the rural group would have at least 80% power to be detected as statistically significant. GLIMMPSE (https://glimmpse.samplesizeshop.org) was used for these calculations.

After data entry, data were checked and cleaned using range and consistency checks. The demographic characteristics of the samples are presented as frequency counts and associated percentages for categorical measures and mean (±SD). The distributions of continuous measures were visually reviewed with the aid of histograms. Distributions were summarized using means and standard deviations if symmetric or with medians and 25th and 75th percentiles if nonsymmetric. Pearson's chi-squared test and the independent samples *t*-test were used to test for evidence of differences between the rural and urban populations on sociodemographic and health outcome variables, respectively. Repeated measures analysis of variance (RMANOVA) was used to test for evidence of variation in PA between place and seasons adjusted by age and gender. Because the activity data were positively skewed, the square root transformation was used to produce data for analysis, which followed a normal distribution. Results were back transformed before reporting in the tables. Within the RMANOVA, the Greenhouse-Geisser test was used to so as to avoid the assumption of sphericity. Subgroup analyses were used to investigate the seasonal variation in PA within the rural and urban groups separately. All *p* values below *α* = 0.05 significance level was considered statistically significant evidence of difference between groups or within groups across seasons. Data were analyzed using SPSS (version 23) statistical software.

## 3. Results

### 3.1. Participants Characteristics

The characteristics of the 162 study participants (97 rural and 65 urban) are described in Table 1: 55% were female; the overall mean age was 35 (SD = ±9) years; 19% of the participants had no schooling; two-thirds owned a house; and 82% owned land. Significant differences were noted between the urban and rural groups for age, education, household, and land ownership. A higher proportion of the urban residents were less than or equal to 30 years of age (43% and 29%, respectively) and a higher proportion of the urban residents had completed high school or higher (70% and 47%, respectively). House and land ownership were much higher for rural than urban residents.

Table 2 shows estimates of median energy expenditure in MVPA MET-minutes per week over 12 months as derived from the PYPAQ. The median total MVPA reported was 4753 MET-min/week with rural residents reporting higher total MVPA than urban residents (5914 MET-min/week vs. 2373 MET-min/week; *p* < 0.001). No significant difference was observed between the total MVPA of males (4602.04 MET-min/week) and females (4825.58 MET-min/week). Total MVPA was derived mostly from the occupation (32%), household (38%), and transport (22%) domains. LTPA (7%) and gardening (1%) were minor components of total PA (data not shown).

There was minimal vigorous intensity PA in all domains except for the transport domain among the rural residents where more than two-thirds of the rural residents reported walking while carrying medium and heavy loads. Although males reported significantly higher MET-min/week for moderate intensity occupation-related activities and leisure activities relative to women, women reported higher MET-min/week in the household domain.

### 3.2. Seasonality in Energy Expenditure

**Figure 1:**
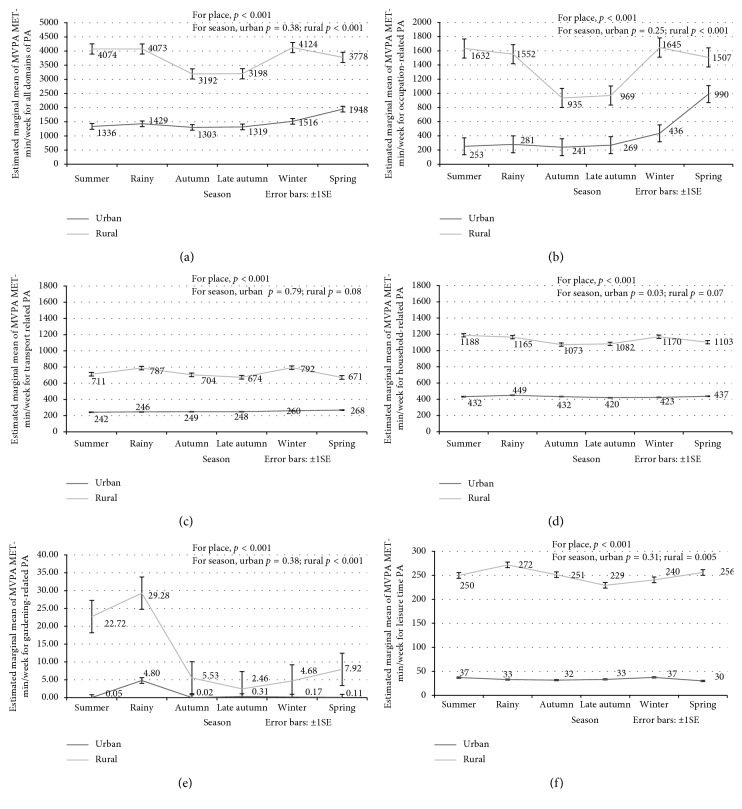
Mean MVPA MET-min/week of 6 seasons of Bangladesh for the urban and rural residents adjusted for age and gender for (a) all domain of physical activity (PA); (b) occupation-related PA; (c) transport-related PA; (d) household-related PA; (e) gardening-related PA; (f) leisure time PA.

Figure 1(a) shows the seasonal variation of MVPA MET-min/week for total PA for the rural and urban residents after adjusting for the potential confounding effect of age and gender. The rest of Figures 1(b)–1(f) shows the equivalent results for each PA domain individually. There were significant differences in total and domain-specific average MVPA MET-min/week between the rural and urban residents in each season (*p* < 0.001). Among the urban residents, seasonal differences were noted only in household-related PA (Figure 1(d), *p*=0.03). By contrast, in the rural residents, statistically significant evidence of seasonal variation was evident for total MVPA (*p*=0.0001) and the occupation (*p*=0.0001), gardening (*p*=0.0001), and leisure time (*p*=0.005) domains, but not for the transport or household domains.

For the rural residents, the overall and occupation-related PA appeared to be highest in the summer, rainy, and winter seasons and lowest in autumn and late autumn. Daily household activities and transport-related PA domains did not show any significant differences between seasons, whereas LTPA and gardening activities average MVPA peaked in the rainy season and decreased to a minimum in late autumn.

### 3.3. Seasonality in Activity Types by Domain

**Figure 2:**
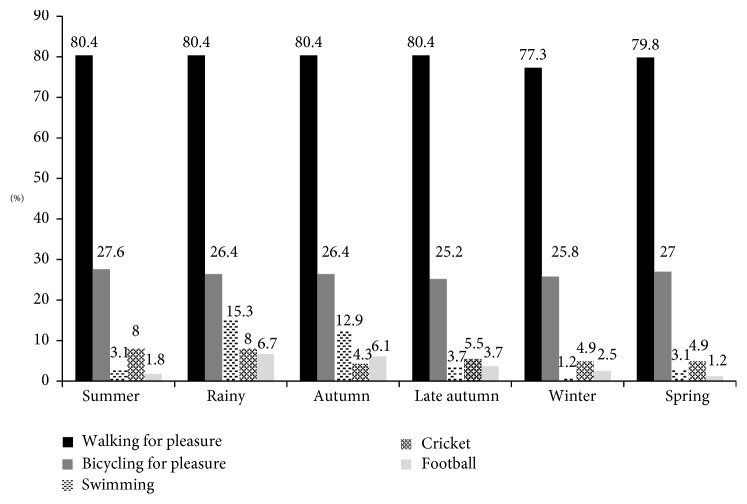
Percentage of participants reporting the most prevalent leisure-time physical activities in Bangladesh.

In the leisure-time domain, each respondent was questioned about 20 separate types of physical activities. However, only five of these activities were recorded by more than 3% of respondents. These more common activities were walking for pleasure followed by cycling for pleasure, swimming, cricket, and football. The relative frequencies for each season are presented in Figure 2. No one reported going to the gym or doing any muscle training and few reported aerobic classes (1.2%). Participation in swimming and, to some extent in football and cricket, seemed to be more common in the rainy and autumn seasons compared to other seasons.

The urban and rural residents showed substantial differences in LTPA participation (data not shown). While 100% of the rural residents participated in walking for leisure, only 50% of the urban residents participated in walking for leisure. Neither group showed seasonal variation in walking for pleasure. Although 41% of the rural residents participated in bicycling for leisure with little seasonal variation, cycling for pleasure was much less common in the urban residents with a noticeable seasonal divide. For the urban residents, bicycling in spring, summer, and the rainy season was reported by 6%, 8%, and 5% of participants, respectively, and by only 2%-3% of respondents in late autumn and winter. Swimming was most common in the rainy and autumn seasons with higher participation in the rural residents (22% and 19%) than in the urban residents (6% and 5%). By contrast, cricket and football were seasonal only in the rural residents: the urban residents played football all seasons (6% participation) except spring (3%), whereas the rural residents played football almost exclusively in the summer, rainy, and autumn seasons (6%).

In the occupational domain, out of 51 activities that were surveyed, 12 showed seasonal changes in participation (data not shown). All of these were related to farming (e.g., poultry work, cleaning the barn, harvesting, processing rice, carrying seeds, etc.) producing the seasonality in occupation-related energy expenditure for the rural residents.

In the transport domain, no activity displayed seasonal variation except of walking while carrying heavy load (>15 kg). This was most common in winter, summer, and rainy seasons (55%, 45%, and 46%, respectively) corresponding to the harvest period (where crops are carried from field to farm or shopping destinations) and less common in autumn or late autumn (∼20%). Similarly, many household activities were stable along the seasons. In the gardening domain, some activities, such as planting trees, were more frequent in the rainy season (37%) as opposed to late autumn and winter (4%) (data not shown).

## 4. Discussion

### 4.1. Main Findings

The questionnaire described in this paper was designed to assess habitual, culturally relevant physical activities in different domains over the past year for working aged men and women residing in Bangladesh. We used this tool to compare the average MVPA of a group of urban residents (from a worksite in Dhaka) and a group of rural residents (from a village in northern Bangladesh; Thakurgaon). Marked differences were found in the patterns of MVPA between the rural and urban residents, with the urban participants reporting much lower levels of energy expenditure than the rural participants on average in every season. Seasonal variations in energy expenditure were statistically significant only among the rural residents. This seasonal variation in PA in the rural residents occurred in most PA domains with the exception of the household and transport domains. Despite relatively comfortable temperature in Thakurgaon during autumn (average 26–28°C) and late autumn (average 18–22°C) [13] when occupational PA of rural residents is low, LTPA did not peak. Prevalent LTPAs in Bangladesh were those that are easily accessible; walking, swimming and bicycling, but the level of participation in these activities was substantially lower in the urban than in the rural residents and among women.

### 4.2. Seasonality

One of the strengths of the PYPAQ is its richness in information; it enables us to capture the variation of PA from season to season and provides information on the most common activity types. When surveillance of PA is based solely on a “usual week” or “the past week,” it can be difficult to detect seasonal variations. It requires continuous and stable data collection over the entire year such as the Behavioral Risk Factors Surveillance (BRFSS) in the USA.

This study is the first year-round surveillance study of PA in Bangladesh and the first description of seasonal variations. Two previous WHO STEPS surveys have collected PA data in Bangladesh. These used the Global Physical Activity Questionnaire (GPAQ) that asked respondents to recall their PA in a “usual week.” The first survey was conducted from November 2009 to April 2010, covering late autumn to spring, with the more recent survey in 2013 covering winter and spring (Jan–March).

We observed statistically significant differences in average energy expenditure between seasons but only among the rural residents and apparently dictated by the agricultural cycles. April-June (summer) is the time for various paddy planting and harvesting duties when farmers are highly engaged with moderate-to-vigorous farming related PA [14]. In contrast, in autumn, while awaiting crops to ripen, there was a significant decline in occupational PA. Similar findings were found in a multisite study of nine Asian rural areas (including Bangladesh) where PA was influenced by the timing of data collection. Specifically, PA level was 20% lower when the survey was conducted during June to October compared to when the survey covered the rainy season, monsoon, and early winter [15]. Surprisingly, the recreational LTPA in the rural residents followed the occupational energy expenditure pattern. Also, LTPA declined in autumn despite average daily temperature being similar to summer when LTPA was high. Similarly, the temperature during the late autumn and winter in Bangladesh was quite pleasant for recreational activities, but the recorded energy expenditure in LTPA was relatively low. While, in western countries, PA was generally found to be lower in winter compared with summer or autumn [16–18], there are exceptions. For example, extreme hot weather, such as in Arizona's summer, was shown to reverse the usual seasonal pattern of the wider USA [5]. In the USA, according to the BRFSS yearlong survey, energy expenditure in spring and summer was 15%–20% higher than in fall and winter. This arose through a combination of higher intensity PA, an increased number of activities, and an increased in duration of participation in each activity [17]. In Bangladesh, the difference between summer and winter temperatures is less extreme and thus less likely to contribute to variation in PA. This may suggest that seasonal variation in PA in the rural residents maybe more strongly related to living the traditional agricultural calendar than to the weather.

In contrast to rural residents, the average MVPA MET-min/week for the urban residents in Bangladesh was quite stable throughout the year in all domains. One explanation could be the urban residents were selected from one workplace where occupation-related PA is stable throughout the year. This is the typical pattern of PA when a person who lives in urban area holds a regular job with similar tasks throughout the year. Other industries and jobs such as construction workers and masons may be more susceptible to changes in the season. Indeed, even the availability of work and number of jobs held could be subject to seasonal variation for some employment categories such as day laborers. It would therefore be worthwhile to assess seasonal variations in PA for a more representative sample of urban resident from a variety of work sites and professions to corroborate the flattened seasonal pattern. A recent study of university students in Dhaka showed that students were more concerned by poor lighting and a lack of convenient places to do PA [19] than weather conditions. Therefore, one reason why the rate of participation in walking or cycling was so much lower in the urban residents may be the poor condition of footpaths, the heavy traffic, and poor lighting [19]. Swimming, in Bangladesh, is an accessible PA with large areas of natural ponds and lakes, but these water-based centers may also be more accessible to the rural rather than the urban residents.

### 4.3. Differences in Energy Expenditure by Domain and Place

Overall, marked differences were found in the patterns of PA between the two study groups. The urban participants had much lower levels of activity than the rural participants, which is consistent with other studies from Bangladesh [2, 3]. A large-scale Indian study, the ICMR-INDIAB study, also showed that the prevalence of physical inactivity was significantly higher in rural areas compared to urban areas (65.0% vs. 50.0%) [20]. Furthermore, typical to low-income countries, we found that the occupation, household, and transport domains were the major contributors to total PA [7, 15, 20]. The very low contribution of LTPA to total PA is of concern particularly for the urban residents. The urban residents reported lower prevalence of participation even in the most accessible form of activity, walking. Moreover, high-intensity activities were rarely recorded and there was no report of any planned exercise. Given the strong evidence linking exercise and fitness to cardio-metabolic health, LTPA should be promoted to all Bangladeshis to maximize their health benefits.

The average total MVPA MET-min/week of this study is comparable with previously published values from Past Year Total Physical Activity Questionnaire (PYTPAQ) in Canada [21] and the EPIC-Norfolk questionnaire [22], although this study showed higher values than those obtained from an Indian PAQ (MPAQ) [23]. All these questionnaires assessed PA across the entire year, but the questionnaires' formats are different. For instance, while our questionnaire has a list of prompted activities, PYTPAQ has an open question format, that is, participants were asked to recall all types of activities they did without prompts. This might lead to under reporting of physical activities that were less recently performed. The PYPAQ estimates were found higher than the GPAQ [3] because the PYPAQ inquires about 12 months of physical activity and we express MVPA MET averaged across the whole year. On the other hand, GPAQ asks about one week of a whole year. This week can be the busiest or laziest week of a particular participant relative to long-term habits [9]. Although our results showed higher MVPA MET-min/week than some of the short-period questionnaires like GPAQ and International Physical Activity Questionnaire (IPAQ) [24, 25], other short-period questionnaires produced quite similar results to ours. These include, for example, the Indian migration study physical activity questionnaire (IMS-PAQ) [26], Multi-Ethnic Study of Atherosclerosis (MESA) questionnaire [27] and the IPAQ conducted in Hong Kong's Chinese population [28]. It is indeed challenging to compare PA between different populations because of the differences in the instruments used and the variable approaches for classifying PA into levels. Mealing et al. [29] and Brownson et al. [30] showed that the prevalence of meeting PA recommendations varied even in the same population when the same data are analyzed by different PA scoring algorithms.

### 4.4. Strength and Limitations

This study is the first to assess seasonal variations across all domains and in both the rural and urban context; however, some caution should be used when generalizing the findings. The urban sample was selected from one worksite and the rural sample from one geographic area. Like any PA questionnaire, PYPAQ has also some limitations. Firstly, we acknowledge the potential for recall bias when recording the frequency and duration of activities that occurred months prior. It was easier for participants to recall their near past PA than their PA in the more distant past. Participants were asked to recall the time they spend on certain activities in hours or minutes; but in rural settings, people are less likely to watch the clock. Secondly, statistically significant results were found in the study by definition of adequate size, and it is possible that there are further differences of practical importance between seasons which we have failed to detect in this study. Furthermore, while literacy rate in Dhaka is higher than in the Thakurgaon rural area (74% vs. 40%), our urban sample overrepresented people from the highest education attainment. This may have introduced differential recall bias, with more accurate reporting in urban area than rural area, and therefore heightened the differences between study groups.

## 5. Conclusions

To our knowledge, this is the first physical activity questionnaire in Bangladesh that is culturally and seasonally specific and reflects long-term participation in PA. Our findings highlight the strong seasonal variation in PA in a group of rural residents of Bangladesh and the much lower level of PA in a group of urban residents in all seasons. As occupation and household-related PA were the major contributors to total PA throughout, we encourage a longitudinal study using PYPAQ to investigate whether these domains alone can provide similar health benefits to the well-established benefit from LTPA and conditioning exercise documented in Western societies. Our results suggest that LTPA should be promoted to all residents in Bangladesh given the low prevalence of participation in this domain. However, the approach must be context and cultural specific [31]; such as the WHO ACTIVE framework [32]. For example, the promotion of LTPA in rural areas when occupational demand is low (i.e., in autumn and late-autumn) could be through repeated mass-participation events at the community level, in line with the active societies framework. By contrast, in urban areas the promotion of a safe and well-maintained environment with open public spaces that provide equitable access may be most needed to sustain higher level of LTPA all along the year. Together, such approaches will promote the health and well-being of Bangladesh residents at different locations.

## Figures and Tables

**Table 1:** Characteristics of the rural and urban residents.

Variables	Total (*n* = 162)	Rural (*n* = 97)	Urban (*n* = 65)	*p*
	*n* (%)	*n* (%)	*n* (%)	
Age				
≤30 years	56 (34)	28 (29)	28 (43)	0.001
31–40 years	66 (41)	35 (36)	31 (48)	
≥41 years	40 (25)	34 (35)	6 (9)	

Gender				
Male	74 (45)	47 (49)	27 (41)	0.42
Female	89 (55)	50 (52)	39 (59)	

Marital status				
Unmarried	17 (10)	10 (10)	11 (17)	0.52
Married	131 (81)	86 (89)	55 (83)	
Others	15 (9)	1 (1)	0 (0)	

Education				
Illiterate	17 (10)	8 (8)	9 (14)	0.0001
Informal education	15 (9)	13 (13)	2 (3)	
Primary school completed	40 (25)	31 (32)	9 (13)	
High school completed	62 (38)	39 (41)	23 (35)	
University level	29 (18)	6 (6)	23 (35)	

Ownership of house				
Owned	108 (66)	96 (99)	12 (18)	<0.001
Rented	47 (29)	0 (0)	47 (73)	
Others	7 (5)	1 (1)	6 (9)	

Ownership of land				
Yes	133 (82)	95 (98)	38 (58)	<0.001
No	29 (18)	2 (2)	27 (42)	

Results are expressed as number (%); *X*^*2*^ test was performed to test for differences between rural vs. urban.

**Table 2:** Descriptive statistics of total and domain-specific physical activity derived from the past year physical activity questionnaire (PYPAQ) in MET-min/week averaged over 12 months.

PYPAQ domains MET-min/week	Total (*n* = 162)	Rural residents (*n* = 97)	Urban residents (*n* = 65)	*p*	Male (*n* = 74)	Female (*n* = 88)	*p*
	Median (Q1; Q3)	Median (Q1; Q3)	Median (Q1; Q3)		Median (Q1; Q3)	Median (Q1; Q3)	
Occupation							
Moderate PA	1426.6 (502.10; 2403.69)	1615.8 (1090.10; 2641.79)	426.7 (0; 2326.34)	<0.001	1921.73 (502.10; 2858.65)	1278.75 (461.97; 2283.14	0.036
Vigorous PA	14.4 (0; 114.73)	78.5 (36.92; 224.42)	0 (0; 0)	<0.001	14.06 (0; 150.00)	21.92 (0; 110.77)	0.60

Household							
Moderate PA	1442.5 (682.00; 2180.32)	1857.6 (1296.17; 2505.94)	643.0 (348.46; 1379.60)	<0.001	937.36 (486.94; 1486.45)	1998.02 (1252.99; 2645.97)	<0.001
Vigorous PA	7.8 (0; 105.14)	23.4 (2.16; 166.15)	0 (0; 15.14)	<0.001	2.16 (0; 11.68)	34.61 (0; 186.92)	<0.001

Gardening							
Moderate PA	4.9 (0; 19.80)	12.3 (3.42; 24.57)	0 (0; 0.22)	<0.001	2.85 (0; 13.77)	5.92 (0; 23.65)	0.388

Travel							
Moderate PA	508.0 (314.48; 720.46)	562.1 (402.23; 802.85)	278.1 (134.08; 562.15)	<0.001	562.15 (343.62; 783.92)	458.31 (281.08; 644.13)	0.159
Vigorous PA	235.1 (22.50; 657.26)	476.0 (265.56; 914.46)	13.8 (0; 71.59)	<0.001	504.52 (21.65; 1123.27)	168.68 (22.50; 366.92)	0.001

Leisure							
Moderate PA	169.6 (56.54; 339.23)	339.2 (169.62; 339.23)	36.3 (0; 113.08)	<0.001	181.15 (102.12; 339.23)	169.62 (2.39; 254.42)	0.005
Vigorous PA	0 (0; 161.54)	6.5 (0; 339.23)	0 (0; 3.04)	<0.001	179.55 (1.08; 592.75)	0 (0; 0)	<0.001

PYPAQ—total MVPA	4753.4 (2977.49; 6382.29)	5914.5 (4647.88; 7161.45)	2373.4 (1080.60; 4217.88)	<0.001	4602.04 (2670.44; 7056.11)	4825.58 (3135.58; 6173.83)	0.541

Results are expressed as median (Q1; Q3); Mann–Whitney *U* test was performed for group difference.

## Data Availability

The data that support the findings of this study are available from the corresponding author upon reasonable request.

## Supplementary Materials

Supplementary Materials Supplementary Figure: rainfall seasonal averages in Thakurgaon and Dhaka.
