# Supplementary material for: Seasonal Variations in Physical Activity Domains among Rural and Urban Bangladeshis Using a Culturally Relevant Past Year Physical Activity Questionnaire (PYPAQ)
Source: J Environ Public Health. 2019 Oct 13;2019:2375474. doi: 10.1155/2019/2375474 (PMC6815625; doi:10.1155/2019/2375474)
Supplement: Supplementary Materials — Supplementary Figure: rainfall seasonal averages in Thakurgaon and Dhaka. [file 2375474.f1.docx]

Supplementary Figure: Rainfalls seasonal averages in Thakurgaon and Dhaka
